# Look beyond Catechol-O-Methyltransferase genotype for cathecolamines derangement in migraine: the BioBIM rs4818 and rs4680 polymorphisms study

**DOI:** 10.1186/s10194-015-0520-x

**Published:** 2015-04-30

**Authors:** Maria Laura De Marchis, Piero Barbanti, Raffaele Palmirotta, Gabriella Egeo, Cinzia Aurilia, Luisa Fofi, Serena Piroso, Cristiano Ialongo, David Della-Morte, Giovanni D’Andrea, Patrizia Ferroni, Fiorella Guadagni

**Affiliations:** Interinstitutional Multidisciplinary Biobank (BioBIM), IRCCS San Raffaele Pisana, Rome, Italy; Department of Cardiovascular, Respiratory, Nephrologic, Geriatric and Anesthesiological Sciences, Sapienza University, Rome, Italy; Headache and Pain Unit, Department of Neurological, Motor and Sensorial Sciences, IRCCS San Raffaele Pisana, Rome, Italy; Telematic University San Raffaele, Rome, Italy; Department of Internal Medicine, Tor Vergata University, Rome, Italy; Department of Systems Medicine, Tor Vergata University, Rome, Italy; Research & Innovation (R&I) srl, Padua, Italy

**Keywords:** COMT, Migraine, Catecholamines, Polymorphism, Disability, SNP, Val 158 Met, Genetics

## Abstract

**Background:**

The study of COMT gene polymorphisms in migraine could be of particular interest since impaired catecholaminergic neurotransmission, namely chronic dopaminergic and noradrenergic hypofunction, is a peculiar migraine trait. In this study, for the first time, we focused on the role of COMT rs4818 genetic variant, the polymorphism most strongly affecting COMT activity, in migraine. This study was conducted in a cohort of carefully clinical characterized Caucasian migraineurs recruited in a specifically dedicated migraine biobank, providing also a replication study on rs4680 polymorphism.

**Findings:**

Genotyping of rs4680 and rs4818 Catechol-O-Methyltransferase gene polymorphisms was performed on 380 unrelated migraine patients, and 132 healthy subjects matched for age, gender and race-ethnicity, with no clinical evidence or family history of migraine or other neurological diseases. The rs4680 and rs4818 genotypic frequencies did not deviate from those expected for a population in Hardy-Weinberg equilibrium and did not correlate with demographics or clinical migraine features, even when considering migraine subtypes such as dopaminergic migraine, menstrual migraine, and menstrually related migraine.

**Conclusions:**

COMT genotype does not influence migraine susceptibility or phenotype, even considering rs4818 polymorphism and peculiar clinical subtypes. This finding prompts to go over COMT to explain catecholamine derangement in migraine, exploring enzymes involved in catecholamines synthesis and catabolism, such as monoamine-oxidase, dopamine beta-hydroxylase, tyrosine-hydroxylase or tyrosine-decarboxylase, among others.

## Introduction

Non-physiological catecholamine levels play a major role in migraine aetiology [1]. Clinical, pharmacological and biochemical evidence point to a chronic dopaminergic and noradrenergic dysfunction in migraine. Of note, some patients show during the attack symptoms related to dopamine receptor stimulation such as yawning, somnolence and vomiting or to reduced sympathetic function such hypotension and, occasionally, syncope [2,3]. The deciphering of the biological basis (*biotype*) of these clinical phenotypes would allow a more precise comprehension of migraine mechanisms and a more tailored migraine treatment.

The Catechol-O-Methyltransferase (COMT) gene [OMIM:+116790], mapping on chromosome 22q11.21, encodes for the major enzyme that degrades catecholamines (dopamine, epinephrine, and norepinephrine). Two COMT transcripts isoforms are produced, leading to the synthesis of a soluble protein (S-COMT), localized in blood and peripheral tissues, and a membrane-bound protein (MB-COMT) concentrated in the brain, mainly in the prefrontal cortex [4,5]. The COMT gene contains several SNPs, some of which result in a marked clinical significance. The rs4680 causes a substitution of a Valine (Val: GTG) to Methionine (Met: ATG) at codon 158 for MB-COMT isoform, and at codon 108 for S-COMT isoform [4,5]. These functional amino acid changes lead to a higher dopamine-degrading activity (H: high) for the Val158 variant and to a lower thermostability and enzymatic activity (L: lower) for the Met158 variant [6-8]. COMT activity is 3 to 4 times higher in Val homozygous compared to Met homozygous genotype carriers [9]. The rs4680 has been related to human pain perception, cognitive phenotypes, psychiatric disorders and variations in brain activation and structure [4,10]. The synonymous polymorphism rs4818 causes a C/G substitution at codon 86 of S-COMT and 136 of MB-COMT, corresponding to a Leucine residue. This genetic variant is related to a haploblock comprising rs6269 (A/G), rs4633 (C/T), rs4818 (G/C) and rs4680 (A/G), which strongly affects the efficiency of protein synthesis based on the specific haplotype, leading to a huge variation in enzyme activity [11]. Three major haplotypes influencing COMT activity and pain sensitivity were identified: a “low pain sensitivity” haplotype (G-C-G-G), characterized by a 11.4 times higher COMT activity compared with the “high pain sensitivity” haplotype (A-C-C-G), and an “average pain sensitivity” haplotype (A-T-C-A), with intermediate COMT activity values [11]. Variability at the rs4818 has been correlated to cognitive functions: the G allele, corresponding to high COMT activity levels and low tonic prefrontal cortex dopamine signalling, has been associated to a less efficient planning and problem solving ability, lower prepulse inhibition levels and working memory performance [12].

The study of COMT polymorphism in migraine has been mainly focused on the rs4680 polymorphism (Table 1) however providing inconclusive results, mainly due to small sampling and poor patients’ clinical characterization [13-21]. Surprisingly, the rs4818 polymorphism, despite its strong influence on COMT activity, has still not been investigated. We also noticed that the combined assessment of rs4680 and rs4818 was sufficient to provide a high degree of informativeness relatively to the overmentioned haplotypes [11].

**Table 1:** **Reported association studies between COMT polymorphisms and migraine (pts: patients)**

**Patients (M/F)**	**Headache subtype**	**Healthy controls (M/F)**	**Mean age (years)**	**Country, Race-Ethnicity**	**Polymorphism**	**Comment**	**Reference**
62 (11/51)	-Migraine with aura (33)	64 (14/50)	-Patients (32 ± 5.2)	Turkey, Asian	rs4680	Association between the Met allele and migraine (P = 0.013) and with family history of migraine (P = 0.003).	Emin Erdal et al., 2001 [13]
	-Migraine without aura (29)						
			-Controls (30.7 ± 6.3)				
982 (346/636)	-Migraine (305)	1468	-Patients (46.8)	Norway, Caucasian	rs4680	No association with migraine.	Hagen et al., 2006 [14]
						Association of the Val/Val genotype with non-migrainous headache in female patients (P = 0.04).	
	-Non-migrainous headache (677)						
			-Controls (55.5)				
219	-Migraine with aura (92)	144	-	Germany Caucasian	rs4680	No association.	Mössner et al., 2006 [15]
	-Migraine without aura (127)						
204	-Chronic daily headache with drug abuse (103)	117	-Patients (range 23-81)	Italy Caucasian	rs4680	No association.	Cevoli et al., 2006 [16]
	-Migraine without aura without drug abuse (101)						
			-Controls (over 40)				
97	Migraine without aura (97)	94	-	Korea Asian	rs4680	Met allele associated with a higher pain intensity of headache (P = 0.001) and nausea/vomiting occurring (P = 0.026).	Park et al., 2007 [17]
263	Migraine without aura (55.9%)	274	-Patients (37 ± 16)	Spain Caucasian	rs2020917	No association.	Corominas et al., 2009 [18]
					rs933271		
					rs1544325		
259	Migraine without aura (61.4%)	287	-Controls (55 ± 18)		rs740603		
					rs740601		
					rs4680		
					rs4646316		
					rs174696		
					rs165774		
					rs9332377		
					rs4485648		
270	Migraine with aura (270)	272	-	Germany Caucasian	rs933271	No association.	Todt et al., 2009 [19]
					rs740603		
					rs4680		
					rs4646316		
					rs165774		
					rs5993889		
1132 (240/892)	-Females with hormonally modulated migraine (464)	608 (258/350)	Patients (range 18-50)	USA, White race ~ 90%	rs4680	Significant association with migraine (P = 0.001) and hormonally modulated migraine (P = 0.03)	Sullivan et al., 2013 [20]
			Controls (range 19-50)				
	-Females with nonhormonally modulated migraine (364)						
198 (40/158)	-1st cohort: Migraine without aura (75)	-	-	Italy Caucasian	rs4680	Significant association between the Val/Val genotype and response to triptans.	Cargnin et al., 2013 [21]
	-2nd cohort: Migraine without aura (111)						
	Migraine with aura (12)						

Therefore, in the present study we sought to study the relationship between COMT rs4818 polymorphism and migraine in a detailed clinical migraine Caucasian population, whose biological samples were stored in a specifically dedicated migraine biobank (Interinstitutional Multidisciplinary BioBank-BioBIM), providing also a replication study on rs4680 polymorphism [22].

## Findings

### Populations

Three hundred-eighty Mediterranean Caucasian unrelated individuals affected by migraine without aura (MwoA, n = 189; M/F = 34/155; mean age 40.3 ± 11.9 yrs), migraine with aura (MwA, n = 65; M/F = 16/49; mean age 39.4 ± 12.6 yrs) or chronic migraine (CM, n = 126; M/F = 14/112; mean age 47.5 ± 13.3 yrs) were consecutively recruited at our Headache and Pain Unit (IRCCS San Raffaele Pisana, Rome) from 01/01/2012 to 31/12/2013 [23,24].

As controls we enrolled 132 healthy subjects (M/F = 57/75; mean age 38.3 ± 12.4 yrs) with no clinical evidence or family history of migraine or other neurological diseases, matched for age, gender and race-ethnicity. Informative and consent forms by the institutional Ethics Committee of San Raffaele Scientific Institute were provided to both groups, together with the permission to obtain blood samples for research purposes. The protocol was approved by the institutional review board at San Raffaele Scientific Institute and have therefore been performed in accordance with the ethical standards laid down in the 1964 Declaration of Helsinki. All subjects gave their informed consent prior to their inclusion in the study.

### Assessment of migraineurs’ clinical characteristics

All migraineurs underwent a complete physical, neurological and fundoscopic examination. Detailed information on patients’ socio-demographic characteristics, lifestyle, migraine features, concomitant diseases and medications were gathered with face-to-face interviews using a semi-structured questionnaire. Migraine features included disease duration, family history of migraine, migraine type (episodic, chronic, MwoA, MwA, pure menstrual, menstrually-related [24]), frequency and duration of attacks, location, quality and intensity of pain, presence of dopaminergic symptoms (assessed by asking: ”Before or during the attack do you present yawning, vomiting or somnolence?), presence of unilateral cranial parasympathetic symptoms, response to triptans (rated as absent/poor, fair, good/excellent), duration of chronic migraine, preventive and acute treatment, presence and duration of medication overuse.

### DNA extraction and genotyping

All biological samples were stored at the BioBIM of IRCCS San Raffaele Pisana following the biobanking Standard Operative Procedures (SOPs) [25]. DNA was isolated from ethylenediaminetetraacetate (EDTA) anticoagulated whole blood using MagNApure LC instrument and the MagNApure LC total DNA isolation kit I (Roche Diagnostics GmbH, Mannheim, Germany) according to the manufacturer’s instructions.

COMT polymorphisms rs4818 and rs4680 were determined by a 261-bp standard PCR amplification in a GeneAmp PCR System 9700 (Applied Biosystems, Carlsbad, CA, USA) using HotStarTaq Master Mix (HotStarTaq Master Mix Kit, QIAGEN, Inc., Chatsworth, CA, USA) on the basis of the COMT Ensembl sequence [Ensembl: ENSG00000093010], using the following primers: F5′-TGTGCTCACCTCTCCTCC-3′ and R5′- CAGGTCTGACAACGGGTC-3′.

All Sanger sequencing analyses were carried out in order to exclude pre-analytical and analytical errors on both strands using Big Dye Terminator v3.1 Cycle Sequencing kit (Applied Biosystems), run on an ABI 3130 Genetic Analyzer (Applied Biosystems) and repeated on PCR products obtained from new nucleic acid extractions.

### Statistics

Data are presented as mean and standard deviation (SD). The allelic frequencies were estimated by gene counting, and the genotypes were scored. The observed numbers of COMT genotypes were compared with that expected for a population in Hardy-Weinberg equilibrium. The significance of the differences of observed alleles, genotypes and haplotypes between groups as well as analysis of multiple inheritance models (codominant, dominant, recessive, overdominant, and log-additive) were also tested using SNPStats (http://bioinfo.iconcologia.net/snpstats/start.htm).

Association of nominal variables was assessed by chi square test of Fisher-Freeman-Halton (FFH) test where needed. For continuous variables, association with a factor was assessed by General Linear Model (GLM) corrected for sex and familiarity for migraine with Tukey’s HSD (Honest Significant Difference) post hoc test. All calculations were made by SPPS 20 (IBM Corp, Armonk, NY), except for power analysis, done by G*Power 3.1.3 free software (Heinrich Heine Universitat, Dusseldorf, Ge). The statistical power reached for GLM for fixed main effects was of 94.5% for an effect size f 0.2 and a type I error probability of 5%.

### Study results

Our study population consists of 380 Caucasian individuals affected by migraine (MwoA = 49,7%, MwA = 17,1%, CM = 33,2%), and 132 healthy subjects. Table 2 reports genotypes and allele frequencies of the rs4818 and rs4680 polymorphisms. No significant differences were found between these frequencies and those predicted by the Hardy-Weinberg equilibrium. Haplotype analysis revealed no linkage disequilibrium between the analyzed polymorphisms. We also tested differences of genotypes between groups by analysis of multiple inheritance models but they were not statistically significant (Table 3).

**Table 2:** **Distributions of genotype and allele frequencies of COMT polymorphism rs4680 and rs4818 observed in patients and controls**

	**N**	**rs4680 genotypes (** ***%*** **)**			**rs4680 alleles (%)**		**HW (P value)**
		**AA (Met/Met)**	**AG (Met/Val)**	**GG (Val/Val)**	**A**	**G**	
Controls	132	23 (17.4)	65 (49.2)	44 (33.3)	111 (42.0)	153 (58.0)	0,90
All patients with migraine	380	78 (20.5)	192 (50.5)	110 (28.9)	348 (45.7)	412 (54.3)	0,73
Migraine with Aura	65	15 (23.1)	30 (46.1)	20 (30.8)	60 (46.2)	70 (53.8)	0,56
Migraine w/out Aura	189	41 (21.7)	96 (50.8)	52 (27.5)	178 (47.1)	200 (52.9)	0,79
Chronic Migraine	126	22 (17.5)	66 (52.4)	38 (30.1)	110 (43.7)	142 (56.3)	0,47
	**N**	**rs4818 genotypes (** ***%*** **)**			**rs4818 alleles (** ***%*** **)**		**HW (P value)**
		**CC (Leu/Leu)**	**CG (Leu/Leu)**	**GG (Leu/Leu)**	**C**	**G**	
Controls	132	30 (*22.7*)	76 (*57.6*)	26 (*19.7*)	136 (*51.5*)	128 (*48.5*)	0,08
All patients with migraine	380	111 (*29.3*)	197 (*51.8*)	72 (*18.9*)	419 (*55.1*)	341 (*44.9*)	0,35
Migraine with Aura	65	21 (*32.3*)	30 (*46.2*)	14 (*21.5* )	72 (*55.4*)	58 (*44.6*)	0,59
Migraine w/out Aura	189	50 (*26.5*)	104 (*55.0*)	35 (*18.5*)	204 (*54.0*)	174 (*46.0*)	0,14
Chronic Migraine	126	40 (*31,7*)	63 (*50.0*)	23 (*18.3*)	143 (*56.7*)	109 (*43.3*)	0,84

Values are given as no. (%). HW: Hardy-Weinberg equilibrium.

**Table 3:** **Differences of genotypes distributions between Migraine groups and multiple inheritance models using the WEB Tool SNPStats (** **http://bioinfo.iconcologia.net/snpstats/start.htm** **)**

**Migraine with Aura**				**Migraine w/out Aura**			**Chronic Migraine**		
**Model**	**rs4680 genotypes**	**OR (95% CI)**	**P-value**	**rs4680 genotypes**	**OR (95% CI)**	**P-value**	**rs4680 genotypes**	**OR (95% CI)**	**P-value**
Codominant	G/G	1.00	0.65	G/G	1.00	0.44	G/G	1.00	0.87
	A/G	1.02 (0.51-2.01)		A/G	1.25 (0.75-2.08)		A/G	1.16 (0.67-2.01)	
	A/A	1.43 (0.62-3.32)		A/A	1.51 (0.79-2.89)		A/A	1.11 (0.53-2.29)	
Dominant	G/G	1.00	0.72	G/G	1.00	0.26	G/G	1.00	0.61
	A/G-A/A	1.12 (0.59-2.13)		A/G-A/A	1.32 (0.81-2.13)		A/G-A/A	1.14 (0.68-1.94)	
Recessive	G/G-A/G	1.00	0.35	G/G-A/G	1.00	0.34	G/G-A/G	1.00	0.97
	A/A	1.42 (0.68-2.95)		A/A	1.31 (0.74-2.32)		A/A	1.01 (0.53-1.93)	
Overdominant	G/G-A/A	1.00	0.68	G/G-A/A	1.00	0.78	G/G-A/A	1.00	0.66
	A/G	0.88 (0.49-1.60)		A/G	1.06 (0.68-1.66)		A/G	1.12 (0.68-1.82)	
Log-additive	---	1.18 (0.77-1.79)	0.44	---	1.23 (0.89-1.70)	0.2	---	1.07 (0.75-1.52)	0.72
**Model**	**rs4818 genotypes**	**OR (95% CI)**	**P-value**	**rs4818 genotypes**	**OR (95% CI)**	**P-value**	**rs4818 genotypes**	**OR (95% CI)**	**P-value**
Codominant	C/C	1.00	0.27	C/C	1.00	0.75	C/C	1.00	0.24
	C/G	0.56 (0.28-1.14)		C/G	0.82 (0.48-1.41)		C/G	0.61 (0.34-1.09)	
	G/G	0.77 (0.33-1.81)		G/G	0.81 (0.41-1.59)		G/G	0.66 (0.32-1.38)	
Dominant	C/C	1.00	0.15	C/C	1.00	0.45	C/C	1.00	0.095
	C/G-G/G	0.62 (0.32-1.19)		C/G-G/G	0.82 (0.49-1.38)		C/G-G/G	0.63 (0.36-1.09)	
Recessive	C/C-C/G	1.00	0.76	C/C-C/G	1.00	0.79	C/C-C/G	1.00	0.79
	G/G	1.12 (0.54-2.32)		G/G	0.93 (0.53-1.63)		G/G	0.92 (0.49-1.71)	
Overdominant	C/C-G/G	1.00	0.13	C/C-G/G	1.00	0.65	C/C-G/G	1.00	0.2
	C/G	0.63 (0.35-1.15)		C/G	0.90 (0.58-1.41)		C/G	0.73 (0.44-1.19)	
Log-additive	---	0.84 (0.54-1.31)	0.45	---	0.89 (0.64-1.25)	0.51	---	0.79 (0.55-1.14)	0.21

We found no association between rs4818 and rs4680 polymorphisms and migraine susceptibility (P = 0.336, and P = 0.577, respectively), migraine type (MwoA, MwA, CM) (P = 0.620, P = 0.858), gender (P = 0.081, P = 0.220), mean age of onset (P = 0.821, P = 0.526), laterality of attacks (P = 0.917, P = 0.954), dopaminergic symptoms (P = 0.615, P = 0.089), unilateral cranial autonomic symptoms (P = 0.233, P = 0.680), migraine prophylaxis (P = 0.489, P = 0.900), analgesics overuse (P = 0.647, P = 0.399), type of analgesic overused (P = 0.800, P = 0368) or other clinical and sociodemographic characteristics (data not shown). No association was found between COMT rs4818 and rs4680 genotypes and pharmacological treatment response, particularly in the 218 patients treated with triptans (P = 0.558, P = 0.956). Furthermore, no correlation was found between rs4818 and rs4680 polymorphisms and pure menstrual or menstrual related migraine (n = 85 pts) (P > 0.05).

## Discussion

The study of COMT gene polymorphisms in migraine is intriguing since an impaired catecholaminergic neurotransmission, namely a chronic dopaminergic and noradrenergic hypofunction, is a peculiar migraine trait [1-3]. This is the first COMT study performed on a cohort of carefully clinical characterized Caucasian migraineurs recruited in a specifically dedicated migraine biobank [23] and focused on the role of rs4818 variant, the polymorphism most strongly affecting COMT activity. In our association analysis the control group was chosen balance the average size of the three pathological groups considered. Therefore, power analysis showed that such a setup sufficed the requirement of at least 90% sensitivity respect to a pretty small effect size (almost comparable to an Odds Ratio of 2.066). In our opinion the power we reached ensured that the negative findings we reported were due to a lack of significant association, rather than to a misrecognized negligible effect.

The main finding of the present study is that COMT rs4818 polymorphism does not influence the susceptibility or clinical phenotype of MwoA, MwA or CM, even when considering migraine patients subsets, such as those characterized before or during the attack by symptoms due to pre- or post-synaptic dopamine receptor activation, such as yawning, vomiting and hypotension (dopaminergic migraineurs) [3] or those with pure menstrual or menstrually related migraine (hormonally modulated migraine, HMM [20]). Moreover, our replication study supports previous results excluding any correlation between COMT rs4680 polymorphism and migraine in Caucasians patients [14-16,18,19], in contrast with a putative correlation found only in Korean and Turkish population [13,17]. Such discrepancies may be related either to smaller sample size or to differences in variant frequency related to race/ethnicity of different population studies.

Our results do not confirm the hypothesis that COMT rs4680 genotype influences the clinical response to triptans as previously suggested [21], albeit our study was performed on a comparable patients sample (218 vs 198 pts). Possible explanations include the fact that our study was not selectively focused on triptans clinical response and considered different criteria to assess triptan responsivity. Finally we did not find significant association between the rs4680 variant and HMM as previously reported by Sullivan et al. [20] probably due to the unequal proportion of women with this subtype of migraine in the present study.

Our study, reasonably, cuts out a direct involvement of COMT gene polymorphisms in migraine even when considering the rs4818 polymorphism. This is not necessarily in contrast with the catecholaminergic derangement widely described in migraine [1]. We suggest that probably other catecholamine catabolic or biosynthetic enzymes, such as monoamine-oxidase, dopamine beta-hydroxylase, tyrosine-hydroxylase or tyrosine-decarboxylase are involved in the dopaminergic and noradrenergic impairment characterizing migraine sufferers [1-3].

Catecholamines originate from tyrosine metabolism through a hydroxylase-related pathway. In migraineurs, a reduction in mitochondrial energy would favour a metabolic shift directing tyrosine metabolism toward decarboxylation pathway instead of the hydroxylation one, leading to reduced catecholamines production and high trace amine levels in the pain matrix, and, ultimately, to the trigemino-vascular system activation, triggering the attack [1].

We acknowledge that potential recall bias and the recruitment in a tertiary-referral center are limitations of our study. Strengths include the use of data based on a specifically dedicated migraine biobank and a careful clinical and demographic characterization of all patients studied using with face-to-face interviews.

## Conclusions

In conclusion, COMT genotype does not influence migraine susceptibility or phenotype, even when considering peculiar clinical subtypes (dopaminergic migraine, HMM). This finding coupled with the well established catecholaminergic imbalance characterizing migraine biotypes prompts to a thorough investigation of other enzymes involved in catecholamines synthesis and catabolism.

## Abbreviations

COMT: Catechol-O-Methyltransferase
S-COMT: Soluble Catechol-O-Methyltransferase
MB-COMT: Membrane bound Catechol-O-Methyltransferase
Val: Valine
Met: Metionine
SNP: Single nucleotide polymorphism
BioBIM: Interinstitutional Multidisciplinary BioBank
SOPs: Standard Operating Procedures
ICHD: International classification of headache disorder
MwoA: Migraine without aura
MwA: Migraine with aura
CM: Chronic migraine
UAs: Unilateral cranial autonomic symptoms
SD: Standard deviation
GLM: General linear model
HSD: Honest significant difference
FFH: Fisher-Freeman-Halton
HMM: Hormonally modulated migraine
NHMM: Non-hormonally modulated migraine
Pts: Patients
